# The role and importance of perceived risk in medical tourism. Applying the theory of planned behaviour

**DOI:** 10.1371/journal.pone.0262137

**Published:** 2022-01-05

**Authors:** Monika Boguszewicz-Kreft, Sylwia Kuczamer-Kłopotowska, Arkadiusz Kozłowski

**Affiliations:** 1 Faculty of Finance and Management, Department of Marketing, WSB University in Gdańsk, Gdańsk, Poland; 2 Faculty of Management, Department of Marketing, University of Gdańsk, Sopot, Poland; 3 Faculty of Management, Department of Statistics, University of Gdańsk, Sopot, Poland; University of Thessaly, GREECE

## Abstract

The main aim of the study was to investigate the role and importance of perceived risk in medical tourism (MT). The research demonstrates that the country of origin and an assessment of the respondents’ own health condition significantly moderates the impact of their attitudes on behavioral intention. The research shows a strong correlation between the risk perceived by the respondents and their attitudes towards MT services. This relationship is significantly moderated by risk awareness, aversion to risk and gender. Additionally, an important factor in the model is the level of satisfaction with medical services in their own country. The survey using a fully structured questionnaire was conducted among young consumers from Jordan, Poland and Turkey. To verify the hypotheses, a multiple regression model with interactions was used.

## Introduction

Participation in medical tourism (MT) may involve a high level of risk as perceived by consumers for several reasons. First of all, this concerns basic human needs such as safety and health [1]. Thus, both medical and tourism services belong to a high-involvement category, which involves significant investments of time, effort and money in purchasing decisions [2–4]. An assessment of MT as a member of the credence products group (in particular, its medical component) is difficult throughout the purchasing process: prior to and during their use and after their consumption [5–9]. The perceived risk is also influenced by factors connected with travelling and staying outside one’s own country, e.g. natural disasters, weather and hygiene problems, diseases, criminality, culture/language barriers and uncertainty with destination-specific regulations and laws, political instability, terrorism etc. [10–18]. Furthermore, MT is a conglomerate of service activities (medical and tourist) with specific characteristics: intangibility, inseparability, variability and perishability, which imply a higher level of perceived risk associated with the purchase than in the case of tangible goods [19–21].

In the scientific discourse the significant attention is paid to the risk in tourism (e.g. [22–24]), however there is little empirical research on this issue in the context of medical tourism. The literature search identified fourteen articles on perceived risks in medical tourism [25–38], including four of a theoretical nature and ten containing the results of primary research (including three using the Theory of Planned Behavior [25,29,32]. (The following e-bases were analysed: EBSCO, ProQuest, Springer, Science Direct, Wiley, and Emerald, using the keywords: "risk perception or perceived risk" AND "medical tourism", filters: scientific journals (scientifically reviewed). This points to a relatively small number of studies on perceived risk in the context of medical tourism. In addition, only two primary surveys included respondents from European countries [27,29].

The Theory of Planned Behavior [39] is one of the most widely used psychological theories in human behavior research [40]. The results of the research conducted to date show that the TPB can also be applied in the MT area [25,29,41–48], however, this thread has so far been related to limited geographical areas and has lacked international comparisons. The authors focused mainly on the markets of East Asia and South-East Asia [25,26,29,42,45–47] and on North American consumers [41,43,44] (The following e-bases were analysed: EBSCO, ProQuest, Springer, Science Direct, Wiley, Scopus, and Web of Science, using the keywords “Theory of planned behaviour” AND “medical tourism”, filters: Scientific journals (scientifically reviewed). However, there are no studies using the TPB in MT in European countries and only one in Middle-Eastern Asia [33], and there are no such comparative analyses between countries. In particular, Khan *et al*. emphasize the scarcity of comparisons between international medical travelers based on their regions of origin, indicating that they may differ in terms of risk perception and intentions [36]. In the context of the mentioned research gap, the authors decided to explore the intentions to use medical tourism services by potential participants from three countries that have not as yet been covered by studies in this aspect (to the best of the authors’ knowledge), namely Poland, Turkey and Jordan, which are important regional markets for medical tourism services [49–52].

The purpose of this article is to investigate the role and importance of perceived risk in medical tourism. To achieve the formulated purpose, the TPB theory, used to examine the intention to use medical services abroad, was verified and extended. The extension consists of adding a component related to the perceived risk of medical tourism and relationship-moderating variables (m.in.: risk awareness, risk aversion and satisfaction with the domestic service, which have not been studied in this context so far) in the basic and extended TPB model. Such an approach is an important contribution to the theory from the point of view of a better understanding of the relationship between risk and the intentions of buyers in medical tourism and the factors that moderate them. The study also fills a research gap concerning three areas in medical tourism, namely a small number of empirical studies on risk, the applicability of the TPB model in this type of activity, and comparisons between countries in general, and between European and Western Asian countries in particular, as it presents the results of the research conducted in Jordan, Poland and Turkey.

Moreover, better understanding of the mechanisms of the impact of risk on purchasing behavior is very important from the point of view of shaping the marketing policy of entities operating on this market.

## Literature review

### TPB: Assumptions

Multiple issues related to the consumer decision-making process are significantly present in the global scientific discourse. Their significance results from the importance of understanding the sources and mechanisms of human behavior and the related decision-making processes for the ability to anticipate, influence and change human actions. A well-documented and rigorously tested theoretical framework for the analyses and prediction of target-oriented human behavior—among the most widely used psychological theories–is the Theory of Planned Behavior (TPB) [41] formulated in 1991 by Ajzen [39]. It assumes the possibility of anticipating and explaining various types of human behavior by identifying their intentions, which are to be predictive and accurate. In accordance with the concept of the TPB, intentions include all and any motivational components of human behavior, and they indicate their predisposition to specific behavior and the cost that people are prepared to incur. In the opinion of the creator of the TPB, intention to engage in a specific action is formed by three groups of factors, whereas the magnitude of the impact of each of them on intentions varies depending on the specific behavior and situation. The factors postulated by the TPB include the following:

- attitude towards behavior, understood as a subjective assessment by an individual—a favorable or unfavorable assessment of the expected effects of a given type of behavior [53]. It should be emphasized that, in contrast to the traditional understanding of attitude as the assessment of a given object, in TPB only behavior is assessed [54]. For example, using services abroad may be perceived as a lack of patriotism, but under certain conditions, such as the lack of appropriate domestic service providers, using them may be assessed positively;
- subjective norms that follow from the social pressure perceived by an individual in relation to the implementation of particular behavior, or refraining from doing so;
- perceived behavioral control over the behavior, described as subjectively assessed ease or difficulty related to specific behavior, resulting from anticipated obstacles and the experience gained so far by an individual (a subjective assessment of the resources and capabilities that are at one’s disposal).

The study conducted in many research areas has shown the validity of TPB in social sciences [54]. It has been used in many different studies that explored health-related behaviors [55–59] and the consumption-related intentions of tourists [60–70].

### The importance of perceived risk in the consumer behavior of international tourists, including medical tourists

Perceived risk is considered to be a focal point for consumer choice, assessment and behavior [71,72]. Bauer (1960) introduced the concept of perceived risk into the literature [73]. This category is defined differently depending on the product/service and the research context [72,74]. Generally speaking, this is an experience of anxiety or psychological discomfort [75] related to potential consequences, long-term adverse impacts and the involuntariness of exposure [11]. It is worth noting that, in contrast to such disciplines as the behavioral decision theory or other areas of psychology, where risk is considered both positively and negatively, in consumer psychology it is associated only with negative consequences [76].

Perceived risk is an important factor that has an influence on travel-related decisions [4,14,17,18,77,78], including intentions [79–81]. However, the study by Quintal and Polczynski (2010) did not confirm this [82], perhaps because it concerned a destination known to the respondents and which they considered safe.

Studies have confirmed that the decisions taken by tourists are influenced more by perceived risk rather than by the actual risks (facts and real circumstances) associated with travelling to specific destinations [83]. “Tourism decisions seem to be made in the heart, not in the head” [83] p.180). In practice, this means that “in marketing, consumer perception is reality” [14], p. 236).

Research on risk perception in international tourism analyzed such factors as: the nationality of tourists [4,23,78]; their cultural dimensions [4,14,23]; religions [75]; personality [16]; destinations [14,84]; gender [4,79]; differences between first-time and repeat visitors [11]; and previous travel experiences [14,17].

Perceived risk is an inhibitor to travel [85,86]. Tourists tend to avoid risky destinations [14,78,85,87], and the perceived risk has an impact on their hesitation and delayed travel decisions [88].

Risks in tourism are related both to the stay in a given destination and to the travel process [80]. In the case of MT, in both aspects, risk perception may be increased when compared to conventional tourists. A vast majority of medical activities involve more or less risk to patients’ health and lives (no expected effects of medical procedures, complications, or side effects). Khan *et al*. (2017a) classified risks related to MT in five groups [36]: 1) Risks related to health at the destination; 2) Risks related to long air travel; 3) Destination related risks; 4) Medico-legal risks; 5) Pre-operative and recuperation risks. In turn [89] divided TM risks into external and internal risks and distinguished the following risk types: financial/economic, psychological, operational, equipment or technical, health, accident/physical, social, time and loss of opportunity risk.

Eight identified studies related to the risk in MT showed the following: differences in risk perception between consumers from different countries and depending on their health status [27]; the link between attitude and perceived risk in general [29] and different dimensions of perceived risk [32]; the link between perceived risk and attitude, subjective norm and perceived behavior control [25]; confirmation of the relationship between perceived risk and purchasing intentions [26] and the lack of such relationship in the research conducted by [37], relationship between perceived risk and perceived value [31]; negative effect of perceived risks on perceived destination image—this image was a strong predictor of visit intention among medical tourists [33]. Two subsequent studies included respondents for whom the potential country of providing medical services was not associated with a language and cultural barrier. The first study concerned diasporic MT [28], where female Korean-born US residents saw the risk of delaying health care and traveling a vast distance associated with additional costs. In the second study, conducted among Chinese residents in the context of using MT in Taiwan, it was found that perceived value was a key predictor of customer intentions, while perceived risk had significant negative influence on the perceived value.

## Research model and hypotheses

The results of the research conducted to date show that the TPB can be applied in the MT area [29,32,41–48]. However, since this thread has so far been related to limited geographical areas and has lacked international comparisons, the authors decided to explore the intentions to use medical tourism services by potential participants from three countries that have not as yet been covered by studies in this aspect (to the best of the authors’ knowledge), namely Poland, Turkey and Jordan.

H1: There is a link between attitude towards participation in medical tourism and the intention to participate in/consider participation in medical tourism.
H2: There is a link between subjective norms and the intention to participate in/consider participation in medical tourism.
H3: There is a link between perceived behavioral control and the intention to participate in/consider participation in medical tourism.

**Fig 1:**
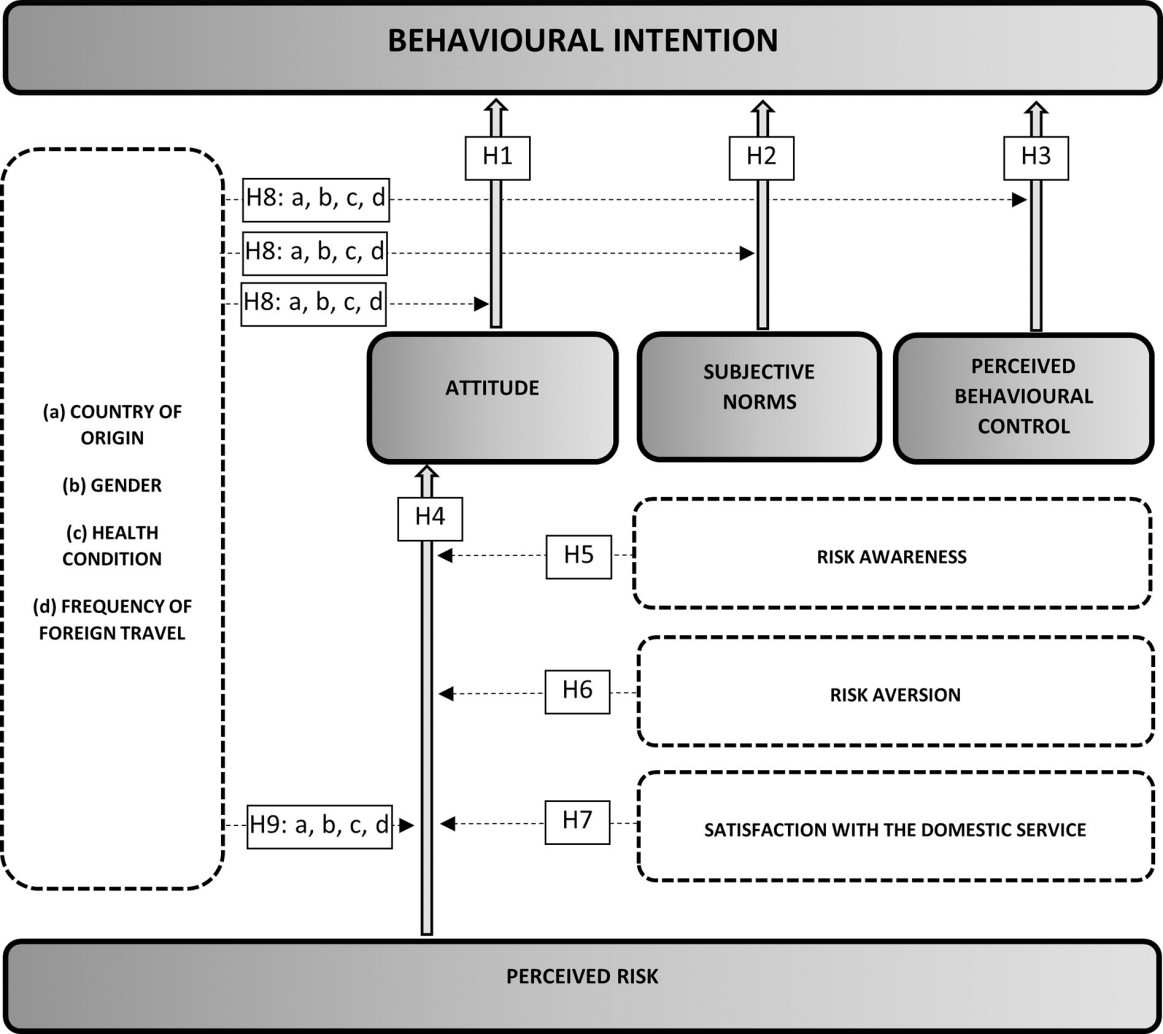
Research model. Source: Author’s own study.

The research was conducted on the grounds of the basic Ajzen TPB model [39], which was extended to include the relationship between the perceived risk and the respondents’ attitude towards medical tourism services (Fig 1). As pointed out by Ajzen, the TPB model is open in principle, and its concept makes it possible to include additional predictors [39]. Such attempts have been successfully undertaken in research on various products and services, in which the model was extended, also in the context of tourism [90], or pro-health behaviors, in which factors such as past behavior [91], attitudes towards advertisement [92], self-concept [93] were taken into account. In the authors’ opinion, the perceived risk related to the use of medical services abroad may constitute such an additional predictor.

Risk is treated as a multidimensional construct [94]. In tourism, there were initially five dimensions of risk specified: financial, performance, physical, social and psychological, which with time in subsequent studies were expended with additional dimensions, such as: equipment, time, satisfaction, opportunity loss, political instability/unrest, health and terrorism, crime, weather and cultural barriers [74].

In this study, perceived risk is understood as psychological discomfort and anxiety [75], and it is considered in terms of cognitive psychology as the subjective perception of negative consequences associated with travel [95].

Previous studies demonstrated that perceived risk may constitute an important factor that has an influence on travel-related consumer decisions [4,14,17,18,78], including intention [79–81,96]. Quintal *et al*. (2010) also demonstrated a negative effect of varying strength by examining the impact of perceived risk on attitude with the use of the TPB [62], similar relations have been shown in the context of MT area [29,32]. In turn, the relations with all the variables in TPB, i.e. attitude, subjective norm and perceived behavioral control, were shown in research [25] conducted in MT in the context of cosmetic medicine. Moreover, the research of [31] has shown a negative relation between perceived risk and perceived value as a key predictor of customer intentions in MT [30].

In this study, the authors decided to test the influence of risk on attitude, similar to [29,32]. Although there are no substantive obstacles for testing the impact of perceived risk on subjective norms and perceived behavioral control as [25], however, in the authors ’opinion it intuitively seems unjustified, and the preliminary data exploration of the authors’ Research shows that perceived risk primarily affects attitude.

H4: There is a link between the perceived risk and attitude towards participating in medical tourism.

Risk perception is a key factor in selecting a tourism destination [97,98]. Due to the fact that the perceived risk is a subjective category, the same situation may be assessed differently in this respect by different people [98,99], it is also shaped depending on the geographical region/country where tourists go, their perception of risk varies [14,84] and tourists tend to avoid risky destinations [14,78,85,87]. It is related with exceeding their subjective acceptable risk threshold [100]. Therefore, there are grounds to assume that *risk awareness* associated with the location of medical procedures abroad (RP) is a moderator of the strength of the relationship between perceived risk and attitude. At the same time, only this type of risk has been included in this study, given that the respondents have not previously used MT. Thus, they might have difficulty in understanding the nature of the services and the associated risks [37], while the sense of risk is related rather to an overall image of the destination.

H5: The level of risk awareness declared by the respondents moderates the relationship between the perceived risk and attitude towards participating in medical tourism.

Risk aversion is associated with an unwillingness to become engaged in behaviors that involve risk [101,102], in other words it is an attitude towards uncertainty [22]. Studies show that risk aversion may influence consumer decision making [103], and Teitler-Regev & Tavor, (2018) proved that „individuals with higher general risk aversion also have higher risk aversion in tourism” [104] (p. 467). However, as indicated by Williams & Baláž (2013), the attitude towards taking risks is the subject of very few studies in the field of tourism [105]. This concept is also relevant in MT because it is a circumstance in which a patient may experience many unfamiliar situations [106]. Studies have confirmed that the perception of risk of tourists varies depending on uncertainty avoidance [4,14], which is a concept related to risk aversion. It was therefore considered to be another factor—besides *risk aversion*—that moderates the strength of the relationship between attitude and the perceived risk.

H6: The level of risk aversion declared by the respondents moderates the relationship between the perceived risk and attitude towards participating in medical tourism.

Despite the fact that the conducted literature studies did not identify similar subject studies by other authors, it can be assumed that dissatisfaction with the level of services provided in one’s own country is a factor that increases the tendency to seek help abroad and that changes the impact of the perceived risk on attitude towards participation.

H7: Satisfaction with the domestic service moderates the relationship between the perceived risk and the attitude towards participating in medical tourism.

Additionally, other variables which potentially moderate relations in the model were taken into account, e.g. the country of origin, gender, health condition, frequency of foreign travel, as well as risk awareness, risk aversion and satisfaction with the domestic service (in the extended model). The conclusions that follow from the existing research indicate differences in attitudes between consumers from different countries towards foreign products and services [107–114]. These were also pointed out in the context of the dimensions of the country of origin of medical tourism services among respondents from Germany, Lithuania and Poland [115]. Taking the above into account, the basic research model was extended to include an additional variable (country of origin), one which potentially moderates the relationship between the variable explained (behavioral intention) and the explanatory variables (attitude, subjective norms and perceived behavioral control). In addition to this, the study also included an attempt to determine whether relations in the basic TPB model differ depending on the declared health condition, frequency of travel abroad and the respondents’ gender, as this possibility had been considered in previous tourism [11,17,18,99] and medical tourism [50,116] related studies.

H8a: The relationships between attitude towards participation in medical tourism, subjective norms, and perceived behavioral control and the intention to participate/consider participation in medical tourism are moderated by the country of origin.
H8b: The relationships between attitude towards participation in medical tourism, subjective norms, and perceived behavioral control and the intention to participate/consider participation in medical tourism are moderated by the gender.
H8c: The relationships between attitude towards participation in medical tourism, subjective norms, and perceived behavioral control and the intention to participate/consider participation in medical tourism are moderated by the health condition.
H8d: The relationships between attitude towards participation in medical tourism, subjective norms, and perceived behavioral control and the intention to participate/consider participation in medical tourism are moderated by the frequency of foreign travel.

Similarly to the basic model, on the level of the extended TPB model, a decision was taken to examine the impact of four identical factors that potentially moderate the relationship between the perceived risk and the attitude.

H9a: The relationship between the perceived risk and the attitude towards participating in medical tourism is moderated by the country of origin.
H9b: The relationship between the perceived risk and the attitude towards participating in medical tourism is moderated by the gender.
H9c: The relationship between the perceived risk and the attitude towards participating in medical tourism is moderated by health condition.
H9d: The relationship between the perceived risk and the attitude towards participating in medical tourism is moderated by the frequency of foreign travel.

## Research method

The quantitative research conducted by the authors of the study concerned the role and importance of perceived risk in medical tourism using the TPB model extended by an additional construct in the form of perceived risk. The survey method was used in the research process. The method of data collection adopted by the authors, the type and scope of data did not require any need for ethical approvals to administer the study.

Three countries were selected for the study, which are defined as important regional markets for medical tourism services (Jordan, Poland and Turkey) [49–52]. The selection of this segment was deliberate: it is this group of consumers (well-educated people, who speak foreign languages and represent an economic status that is relatively higher than the average), who are currently potentially less interested in these types of services, but may constitute an important segment of future market participants. The sample of the respondents was selected as a convenience sample of student groups. There were 406 (voluntary and anonymous) subjects in total: 131 from Jordan, 181 from Poland, and 94 from Turkey.

The research tool used to verify the hypotheses was the fully structured questionnaire, in its basic version prepared in English and then translated into three language versions, according to the nationality of the analyzed segments. The process of linguistic validation of the research tool was carried out taking into account the principle of cultural adaptation in order to obtain the possibility of comparing the obtained results in the intercultural dimension [117]. The substantive correctness of the questionnaire was supervised by an expert panel, which included an expert in the field studied, a translator developing a draft document and experts experienced in developing research instruments.

To measure each concept, this study used the following measurement items that had been proven to be reliable and valid in previous studies (all measurement items were slightly revised to fit the context of this study).

As in Reddy *et al*. (2010, pp. 515–516), the research questions included in the questionnaire concerned the intention to consider medical tourism and not its actual use [44].

The respondents were asked the following question about their intention to use medical tourism services: “If travelling abroad to obtain medical treatment were possible, I would consider it” (a 7-point Likert scale from -3 = definitely no, to +3 = definitely yes). Attitude towards participating in medical tourism was determined on the basis of the following question: “Going abroad to get medical treatment in my opinion is …”, which presented possible answers in the form of a semantic differential (-3 = harmful, +3 = beneficial; -3 = unpleasant, +3 = pleasant; -3 = bad, +3 = good; -3 = wrong, +3 = right; Cronbach’s alpha for this scale was 0.81). The final measure of attitude was the average of these four items. Subjective norms were evaluated using the question: “The majority of the people who are important to me would approve of my travelling abroad to get medical treatment” (a 7-point Likert scale from -3 = definitely no, to +3 = definitely yes). Direct perceived control was evaluated using the question: “If I could consider going abroad to get medical treatment, I would be able to do it” (a 7-point Likert scale from -3 = definitely no, to +3 = definitely yes).

The perceived risk has been operationalized as: “Going abroad to get medical treatment in my opinion is …” (a 7-point Likert scale from -3 = risky, to +3 = safe). To examine the general level of consumer aversion to risk in purchasing decisions, similarly to Bao et al. (2003), the respondents were asked to comment on the following statement: “I most often prefer to stick with the services/products I usually buy rather than to try new ones that I’m not sure of” [103]. Risk awareness was measured using the following question: “To what extent would risk awareness associated with medical procedures abroad be important to you when choosing a place for the provision of foreign medical services abroad?” (a 7-point Likert scale from -3 = totally unimportant to +3 = very important). Satisfaction with the level of domestic service was measured as follows: “Are you satisfied with the medical services in your country?” (a 7-point Likert scale from -3 = definitely no to +3 = definitely yes). Health condition was self-assessed by the respondent on a scale from -3 = definitely bad to +3 = definitely good. The question concerning frequency of foreign travel covered the following options: less than once a year, once a year, between twice and five times a year, and more than five times a year.

## Ethics approval and consent to participate

The study concerned the attitudes and opinions of the respondents and they were notified about the purpose of the research before it started. Study participants were asked to fill in the self-assessment questionnaires, no sensitive data was collected. Verbal informed consent was acquired from the participants before they filled out the questionnaire and they had the right to withdraw from the study at any time. The respondents voluntarily participated in this study and their anonymity and confidentiality of information were ensured. Names of participants were not included in the study questionnaire to ensure complete anonymity. All obtained data was completely confidential and special codes were used to ensure anonymity. The data was only used for the research purpose. There was no access to data by individuals or organizations other than the investigators. All procedures performed in this study involving human participants were in accordance with Helsinki Declaration ethical standards and its later amendments or comparable ethical standards. In accordance with institutional requirements of the WSB University concerning the formal ethical approval process, the study and method of informed consent were verbally approved by the Coordinator of the Management Discipline, acting as an ethics committee at the WSB University. In case the consent needs to be documented, the WSB University may issue a written confirmation.

## Data analysis and results

Tables 1 and 2 present descriptive statistics and frequency tables for the variables measured in the study. The distributions of the four variables from the basic TPB model (behavioral intention, attitude, subjective norms, and perceived behavioral control) are similar, with an average of slightly more than 1, standard deviation of approx. 1.5 and moderate left-hand asymmetry.

**Table 1:** Descriptive statistics for quantitative variables.

Variable:	Mean	Std. dev.	Skewness	Min	Max
Behavioral intention	1,19	1,59	-0,53	-3	3
Attitude	1,12	1,15	-0,18	-3	3
Subjective norms	1,09	1,52	-0,52	-3	3
Perceived behavioral control	1,17	1,51	-0,53	-3	3
Perceived risk	-0,79	1,38	0,18	-3	3
Risk awareness	1,14	1,61	-0,56	-3	3
Risk aversion	0,30	1,60	-0,24	-3	3
Satisfaction with the domestic service	-0,05	1,52	-0,15	-3	3
Health condition	0,33	1,99	-0,16	-3	3

Source: Authors’ own calculations based on survey results.

**Table 2:** Frequency tables for categorical variables.

Variable	*Category*	Count	Percent
Gender	*female*	181	45%
	*male*	225	55%
Frequency of foreign travels	*less than once a year*	180	44%
	*once a year*	126	31%
	*from 2 to 5 times a year*	80	20%
	*more than 5 times a year*	20	5%
Nationality	*Poland*	181	45%
	*Turkey*	94	23%
	*Jordan*	131	32%
Age group	*up to 21*	203	50%
	*21–30*	178	44%
	*30 and above*	25	6%

Source: Authors’ own calculations based on survey results.

Since most of the components of the proposed research model are measured directly (by means of a single question) and the model assumes the existence of interactions (both between quantitative variables and between quantitative and qualitative variables), a multiple regression model was used in the analysis [118].

In order to verify the hypotheses, two multiple regression models were estimated: the first model for the verification of hypotheses H1, H2, H3, H8 (a,b,c,d) (hereinafter referred to as TPB-M1) and the second model for the verification of hypotheses H4, H5, H6, H7, H9 (a,b,c,d) (hereinafter referred to as TPB-M2).

In the TPB-M1 model, *behavioral intention* was the dependent variable, and the independent variables included the components of the basic TPB model (*attitude*, *subjective norms*, and *perceived behavioral control*) and the variables which potentially moderate the relationships in the basic TPB model, i.e. *country*, *gender*, *health condition*, and *frequency of foreign travel*. In order to detect the moderation effect, both the main effects and the interactions between the explanatory variables were included in the model. Due to a large number of interactions and possible variants of qualitative variables, the model obtained is complex (it contains many predictors). Some of these predictors do not significantly affect the model, and their presence in the model may adversely affect an assessment of the most important effects; therefore, the model was reduced using stepwise elimination of variables in accordance with the Akaike Information Criterion (AIC). Table 3 presents the estimated regression coefficients (including the results of Student’s t significance tests) for both the complete and reduced models.

**Table 3:** Results of the TPB-M1 model (dependent variable: *Behavioral intention*).

	Full model (Adj. R^2^ = 0.343)				Reduced model (Adj. R^2^ = 0.351)			
	Coef	S.E.	t	p	Coef	S.E.	t	p
(Intercept)	0.549	0.242	2.27	0.024	0.371	0.150	2.48	0.013
Attitude	0.580	0.186	3.12	0.002	0.616	0.117	5.29	0.000
Subjective norm (SN)	0.019	0.138	0.14	0.890	0.179	0.077	2.31	0.021
Perceived behavioral control (PBC)	0.257	0.145	1.77	0.078	0.152	0.052	2.93	0.004
Turkey	-0.183	0.298	-0.61	0.541	-0.151	0.280	-0.54	0.591
Jordan	-0.344	0.281	-1.23	0.221	-0.368	0.260	-1.42	0.157
Male	-0.164	0.202	-0.81	0.416	x	x	x	x
Health condition	0.011	0.063	0.17	0.864	-0.003	0.060	-0.04	0.966
Travel: once a year	-0.276	0.226	-1.22	0.223	x	x	x	x
Travel: 2–5 times a year	0.212	0.316	0.67	0.503	x	x	x	x
Travel: >5 times a year	-0.172	0.522	-0.33	0.742	x	x	x	x
Attitude * Turkey	-0.402	0.205	-1.96	0.050	-0.499	0.178	-2.81	0.005
Attitude * Jordan	-0.088	0.198	-0.44	0.657	-0.129	0.177	-0.73	0.465
Attitude * Male	-0.122	0.135	-0.90	0.369	x	x	x	x
Attitude * Health condition	0.086	0.042	2.08	0.038	0.099	0.038	2.60	0.010
Attitude * Travel: once a year	0.183	0.164	1.12	0.265	x	x	x	x
Attitude * Travel: 2–5 times a year	-0.036	0.196	-0.18	0.853	x	x	x	x
Attitude * Travel: >5 times a year	0.170	0.320	0.53	0.595	x	x	x	x
SN * Turkey	0.339	0.163	2.07	0.039	0.226	0.118	1.92	0.056
SN * Jordan	0.193	0.160	1.20	0.229	0.003	0.111	0.03	0.978
SN * Male	0.193	0.110	1.75	0.081	x	x	x	x
SN * Health condition	-0.030	0.035	-0.86	0.388	x	x	x	x
SN * Travel: once a year	-0.042	0.131	-0.32	0.752	x	x	x	x
SN * Travel: 2–5 times a year	-0.131	0.147	-0.89	0.374	x	x	x	x
SN * Travel: >5 times a year	-0.126	0.224	-0.56	0.574	x	x	x	x
PBC * Turkey	-0.132	0.161	-0.82	0.413	x	x	x	x
PBC * Jordan	-0.235	0.154	-1.52	0.129	x	x	x	x
PBC * Male	-0.101	0.113	-0.89	0.373	x	x	x	x
PBC * Health condition	-0.036	0.034	-1.06	0.291	-0.066	0.025	-2.65	0.008
PBC * Travel: once a year	0.038	0.137	0.28	0.783	x	x	x	x
PBC * Travel: 2–5 times a year	0.184	0.149	1.24	0.215	x	x	x	x
PBC * Travel: >5 times a year	0.231	0.205	1.13	0.260	x	x	x	x

Source: Authors’ own calculations based on survey results.

In the reduced model, the following variables: *gender*, *frequency of foreign travel* (and thus all interactions containing these variables), the interactions between *subjective norms* and *health condition* as well as between *perceived behavioral control* and *country* were removed. The removed variables proved to have no impact on *behavioral intention*, either as the main effect or through interactions with other variables.

In the TPB-M1 model, three explanatory variables from the basic TPB model and two moderating variables (*country* and *health condition*) remained. An assessment of the coefficients of the model is not obvious, as each explanatory variable interacts with at least one of the other explanatory variables. The regression coefficient for the main effect exhibits the effect of a given independent variable on a dependent variable, provided that the variables that interact with this independent variable are on reference levels. The reference levels for qualitative variables are those variants that are not included in Table 3 (i.e. Poland for *country*, female for *gender*, less than once a year for *frequency of foreign travel*), while for quantitative variables, the reference level is 0 (the center of scale).

For example, the coefficient for *attitude* in the reduced model is 0.616. This means that an increase in *attitude* by one unit will result in an increase in *behavioral intention* of 0.616 units on average, with other variables remaining unchanged and provided that the variables interacting with *attitude* are at the reference level. Therefore, this relationship applies to Poland and to those assessing their *health condition* as 0 (average). In order to obtain an estimate of the impact of *attitude* on *behavioral intention* in another country, the coefficients for the main effect and for the interaction of *attitude* with that country need to be added together. For example, for Turkey, the impact of *attitude* on *behavioral intention* is 0.616–0.499 = 0.117, which is much weaker than for Poland, yet still positive.

For the *health condition* moderating variable, an interpretation of the interaction is more difficult than for the *country* variable because this is a quantitative variable. For example, the regression coefficient for the interaction of *attitude* and *health condition* in the reduced model is 0.099, which means that as the assessment of *health condition* increases by one unit, the effect of *attitude* on *behavioral intention* increases by 0.099 units on average. For example, for the *health condition* = 1, an increase in the *attitude* by one unit will increase the *behavioral intention* by 0.616 +1 ∙ 0.099 = 0.715 units on average. Or, for *health condition* = -2, an increase in *attitude* by one unit will result in an increase in *behavioral intention* by 0.616–2 ∙ 0.099 = 0.418 units on average (each time, assuming other variables remain at the same level).

In order to supplement the assessment of the impact of the individual explanatory variables and the moderators in the TPB-M1 model, an analysis of variance was carried out to evaluate the total impact of the individual variables on the explained variable. Table 4 presents five types of F tests (see: [119]):

- test for the overall significance of the variable (“main effect + interactions”)
- test for the significance of the interactions alone of a given variable (“All interactions”)
- tests for individual interactions
- test for the total significance of all the interactions (“Total interactions”)
- test for the total significance of the model (“Total”)

**Table 4:** Results of F tests for the joint effect of predictors (main effect + interactions) for the TPB-M1 model (reduced; d.f.–degrees of freedom, Partial SS–partial sum of squares, MS–mean squares, F–F-statistics, p–p-value).

	d.f.	Partial SS	MS	F	p
Attitude (main effect + interactions)	4	121.6	30.4	18.6	0.000
All Interactions	3	22.4	7.5	4.6	0.004
SN (main effect + interactions)	3	45.8	15.3	9.4	0.000
All Interactions	2	7.6	3.8	2.3	0.099
PBC (main effect + interactions)	2	21.1	10.6	6.5	0.002
All Interactions	1	11.4	11.4	7.0	0.008
Country (main effects + interactions)	6	32.7	5.5	3.3	0.003
All Interactions	4	19.2	4.8	2.9	0.020
Health condition (main effect + interactions)	3	17.6	5.9	3.6	0.014
All Interactions	2	17.1	8.6	5.2	0.006
Attitude * Country	2	16.2	8.1	5.0	0.007
Attitude * Health condition	1	11.0	11.0	6.8	0.010
SN * Country	2	7.6	3.8	2.3	0.099
PBC * Health condition	1	11.4	11.4	7.0	0.008
Total interactions	6	31.2	5.2	3.2	0.005
Total	12	376.7	31.4	19.2	0.000
Error	393	640.9	1.6	x	x

Source: Authors’ own calculations based on survey results.

In addition to this, the value of F statistics may be interpreted as the importance of a given component, i.e. the strength of its impact on the explained variable.

The following conclusions can be drawn based on the analysis of the results of the TPB-M1 model (Tables 3 and 4):

- Three explanatory variables from the basic TPB model (*attitude*, *subjective norms*, and *perceived behavioral control*) are statistically significant, with a positive effect on *behavioral intention* (the result being compliant with H1-H3), with the *attitude* variable having the strongest impact.
- The interactions occurring in the model have a statistically significant impact on the explanation of the dependent variable (p-value = 0.005; partial compliance with H8).
- *Country* and *health condition* variables moderate the effect of *attitude* on *behavioral intention* in a statistically significant manner. In the countries which are the subject of research, the effect of *attitude* varies, but it is always positive. A higher assessment of *health condition* strengthens the impact of *attitude* on *behavioral intention*.

- *Health condition* moderates the impact of *perceived behavioral control* on *behavioral intention* in a statistically significant manner. A higher assessment of *health condition* weakens the impact of *perceived behavioral control* on *behavioral intention*.
- Some differences can be observed between the countries as regards the impact of the *subjective norms* variable on *behavioral intention*, yet in total they are not very pronounced (the p-value for the test for the total significance of the interaction of *subjective norms* with *country* is 0.099).
- The estimated model explains a small portion (35.1%) of the variability of the *behavioral intention* to use medical tourism.

In the second model of multiple regression (TPB-M2), *attitude* was a dependent variable, while independent variables included *perceived risk*, and potential moderating variables included: *risk awareness*, *risk aversion*, *satisfaction with the domestic service*, *country*, *gender*, *health condition*, and *frequency of foreign travel*. As in the case of the first model, the complete model was estimated, and subsequently the stepwise elimination of variables according to the AIC was performed. The results of the two versions of the model are presented in Table 5.

**Table 5:** Results of the TPB-M2 model (dependent variable: *Attitude*).

	Full model (Adj. R^2^ = 0.404)				Reduced model (Adj. R^2^ = 0.409)			
	Coef	S.E.	t	p	Coef	S.E.	t	p
(Intercept)	0.595	0.138	4.30	0.000	0.633	0.125	5.05	0.000
Perceived risk	-0.456	0.087	-5.27	0.000	-0.438	0.053	-8.21	0.000
Risk awareness	0.159	0.034	4.65	0.000	0.158	0.034	4.66	0.000
Risk aversion	-0.049	0.034	-1.45	0.148	-0.052	0.033	-1.55	0.121
Satisfaction with the domestic service	-0.124	0.035	-3.56	0.000	-0.138	0.030	-4.59	0.000
Turkey	0.110	0.165	0.67	0.505	0.033	0.125	0.26	0.793
Jordan	0.365	0.154	2.36	0.019	0.216	0.109	1.99	0.047
Male	-0.161	0.109	-1.49	0.138	-0.174	0.106	-1.64	0.101
Health condition	-0.034	0.034	-0.98	0.327	x	x	x	x
Travel: once a year	-0.364	0.122	-2.99	0.003	-0.305	0.107	-2.85	0.005
Travel: 2–5 times a year	0.106	0.161	0.66	0.509	0.203	0.128	1.59	0.113
Travel: >5 times a year	-0.174	0.308	-0.56	0.573	-0.126	0.222	-0.57	0.571
Perceived risk * Risk awareness	0.062	0.020	3.04	0.003	0.063	0.020	3.17	0.002
Perceived risk * Risk aversion	-0.049	0.018	-2.75	0.006	-0.050	0.018	-2.86	0.004
Perceived risk * Satisfaction with the domestic service	0.016	0.019	0.80	0.425	x	x	x	x
Perceived risk * Turkey	0.071	0.101	0.70	0.483	x	x	x	x
Perceived risk * Jordan	0.135	0.100	1.35	0.178	x	x	x	x
Perceived risk * Male	-0.181	0.070	-2.59	0.010	-0.185	0.066	-2.81	0.005
Perceived risk * Health condition	-0.026	0.021	-1.21	0.226	x	x	x	x
Perceived risk * Travel: once a year	-0.076	0.085	-0.89	0.374	x	x	x	x
Perceived risk * Travel: 2–5 times a year	-0.080	0.099	-0.81	0.416	x	x	x	x
Perceived risk * Travel: >5 times a year	-0.036	0.168	-0.21	0.830	x	x	x	x

Source: Authors’ own calculations based on survey results.

As a result of the stepwise elimination of the variables, the *health condition* variable and a majority of the interactions were completely removed from the model. *Perceived risk*, *risk awareness*, *risk aversion* and *gender* (the main effects and interactions) and *satisfaction with the domestic service*, *country*, and *frequency of foreign travel* (as the main effects only) remained. As before, an analysis of variance was conducted for a more complete assessment of the model and to facilitate interpretation, the results of which are presented in Table 6.

**Table 6:** Results of F tests for the joint effect of predictors (main effect + interactions) for the TPB-M2 model (reduced).

	d.f.	Partial SS	MS	F	p
Perceived risk (main effect + interactions)	4	176.3	44.1	56.2	0.000
All Interactions	3	16.9	5.6	7.2	0.000
Risk awareness (main effect + interactions)	2	17.4	8.7	11.1	0.000
All Interactions	1	7.9	7.9	10.1	0.002
Risk aversion (main effect + interactions)	2	6.4	3.2	4.1	0.017
All Interactions	1	6.4	6.4	8.2	0.004
Satisfaction with the domestic service	1	16.5	16.5	21.0	0.000
Country	2	3.4	1.7	2.2	0.116
Gender (main effect + interactions)	2	6.2	3.1	4.0	0.019
All Interactions	1	6.2	6.2	7.9	0.005
Travel	3	13.5	4.5	5.8	0.001
Perceived risk * Risk awareness	1	7.9	7.9	10.1	0.002
Perceived risk * Risk aversion	1	6.4	6.4	8.2	0.004
Perceived risk * Gender	1	6.2	6.2	7.9	0.005
Total interactions	3	16.9	5.6	7.2	0.000
Total	13	227.9	17.5	22.4	0.000
Error	392	307.2	0.8	x	x

Source: Authors’ own calculations based on survey results.

On the basis of an analysis of the results of the TPB-M2 model (Tables 5 and 6), the following conclusions can be drawn.

*Perceived risk* has a very strong negative impact on *attitude* (the higher the *perceived risk*, the less favorable the *attitude* to medical tourism; this conclusion is in line with H4), assuming that the variables that interact with *perceived risk* remain at level 0 (the middle level).

Both *risk awareness* and *risk aversion* significantly moderate the relationship between *perceived risk* and *attitude* (compliance with H5-H6), with *risk awareness* being the stronger moderator. Additionally, *gender* also acts as a moderating variable between *perceived risk* and *attitude* (partial compliance with H9b).

**Fig 2:**
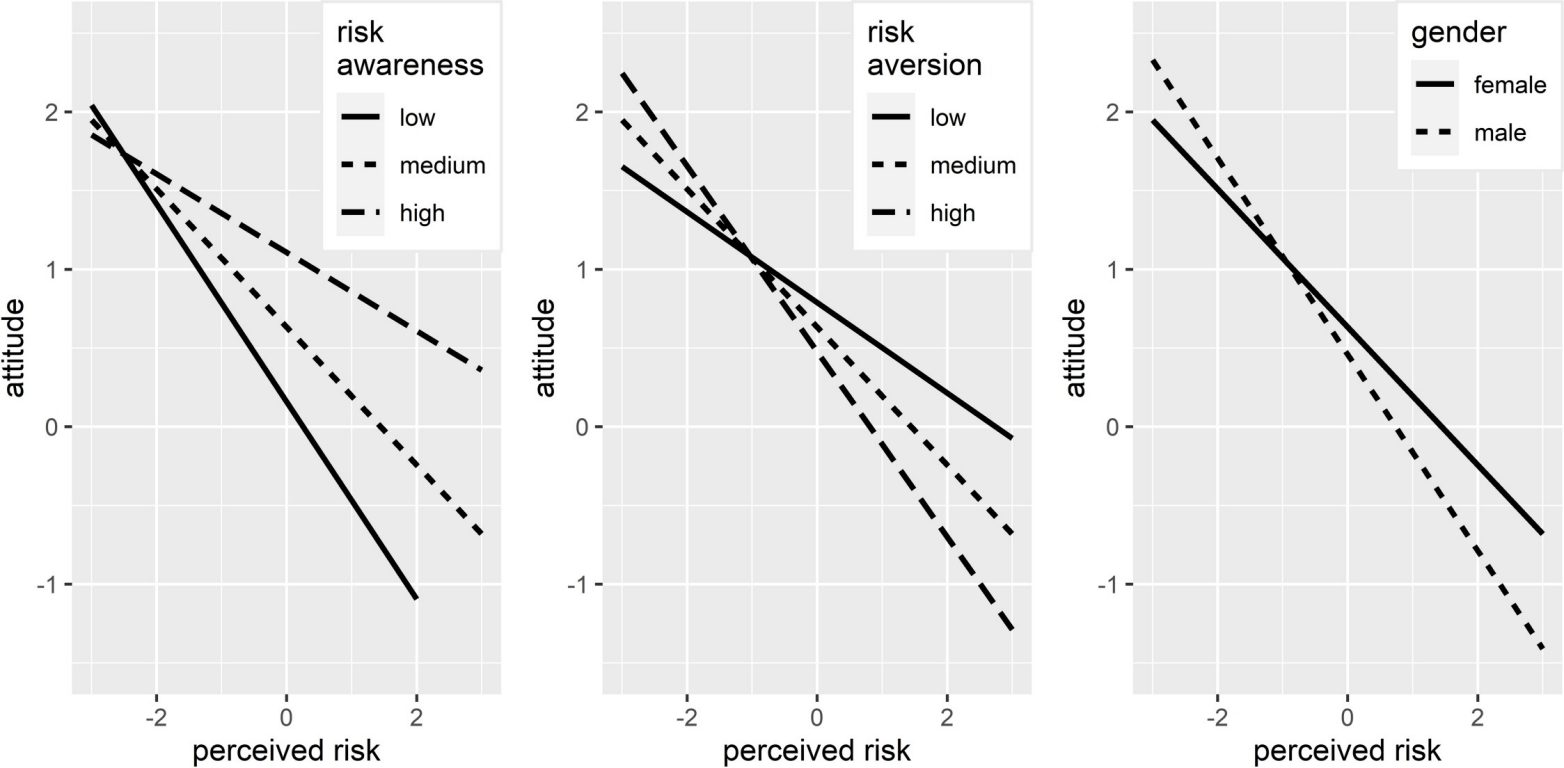
Theoretical relationship between *perceived risk* and *attitude* for different values of *risk awareness*, *risk aversion*, and *gender* (with other variables at reference levels). Source: Authors’ own calculations based on survey results.

In order to assess the nature of these moderations, it is necessary to take a look at both the regression coefficient with the moderating variable and the interaction of this moderating variable with *perceived risk*. To facilitate this assessment, lines that represent the estimated relationship between *perceived risk* and *attitude* are placed in Fig 2 for the different values of the moderating variables (the remaining variables in the model, which are not included in the graph, are at reference levels; no change in these levels affects the slope of the lines presented in the graph).

The impact of *risk awareness* on the relationship between *perceived risk* and *attitude* is as follows. The higher the level of *risk awareness*, the weaker the relationship between *perceived risk* and *attitude* (the slope of the regression line is not as steep). This is because the coefficient by interaction (0.063) is positive, and the coefficient with the *perceived risk* is strongly negative (-0.438). In addition to this, due to the high value of the coefficient by the main effect of the *risk awareness* (0.158), the regression lines for higher values of *risk awareness* are above the lines for lower values. In other words, the higher the level of *risk awareness*, the smaller the negative impact of *perceived risk* on *attitude*.

The impact of *risk aversion* on the relationship between *perceived risk* and *attitude* is as follows. The greater the aversion to risk, the steeper the negative slope of the regression line (being the result of the negative coefficient by interaction, -0.050), i.e. the stronger the negative impact of *perceived risk* on *attitude*. In other words, respondents with higher levels of *risk aversion* have a less favorable *attitude* to medical tourism if they perceive it as risky compared to less risk-averse respondents who also perceive medical tourism as risky.

In the case of *gender*, the moderating effect is that the impact of *perceived risk* on *attitude* is stronger among men than women (a steeper negative slope of the regression line). In other words, men who perceive medical tourism as risky are less favorably disposed towards it than women who have a similar perception.

The impact of moderating variables is statistically significant; however, it does not change the direction of the relationship between *perceived risk* and *attitude* (this is always strongly negative), but merely weakens or strengthens this relationship. The *perceived risk* variable also has the strongest impact on *attitude* from among all the variables included in the model (the highest value of F statistics = 56.2, which is much higher than the other values). *Satisfaction with the domestic service* (F = 21.0) proved to be the second most important variable in the model; the relationship is negative (the higher the level of satisfaction with the domestic medical service, the less favorable the attitude to medical tourism); this variable occurs in the reduced model as an additive component only, without any interaction with *perceived risk*. The variables *country* and *frequency of foreign travel* are also present in the reduced model as additive components, yet they have no significant impact on *attitude* (F statistics equal to 2.2 and 5.8 respectively). In the TPB-M2 model, similarly to the TPB-M1 model, less than half of the variability of the dependent variable (40.9%) is explained by the estimated model.

## Discussion

The results of the study demonstrated a significant impact of all three explanatory variables from the basic TPB model (attitude, subjective norms and perceived behavioral control) on the intention to use medical tourism services at a similar level to other studies [32] which were also conducted with young respondents (students) [41]. Nevertheless, the strength of the impact of individual dependent variables was only partially consistent with the results provided by other researchers.

By analyzing the aggregate results obtained from the respondents from all three countries examined, attitude proved to be the strongest predictor, as in Martin *et al*. (2011), Reddy *et al*. (2010), Seow *et al*. (2016), Dash (2021) and Liang et al. (2019) [25,32,41,44,45]. Other studies that analyzed the role of attitude confirmed its relationship with selected aspects of medical tourism; however, the results did not indicate that it had the strongest impact [42,47]. Only the study conducted by Ramamonjiarivelo *et al*. (2015) did not confirm its relevance [43].

In this study, similarly to those of Martin *et al*. (2011), Reddy *et al*. (2010) and Seow *et al*. (2016) [41,44,45], subjective norms constituted the second most powerful impact on the endogenous variable. In the studies by Lee *et al*. (2012) and Ramamonjiarivelo *et al*. (2015) [43,48], these possessed the highest impact strength, in the research by Liang et al. (2019) [25] the highest impact along with attitude, while in the study by Suki *et al*. (2017) and Dash (2021) this factor proved to be insignificant [32,47].

The impact strength of the perceived behavioral control was the lowest, which is also evident in the results of studies by Martin *et al*. (2011), Ramamonjiarivelo *et al*. (2015) and Liang et al. (2019) [25,41,43]. By contrast, this factor was the strongest predictor in the studies by Suki *et al*. (2017) [47] and the second most important in the research Dash (2021), while the results of the studies conducted by Seow *et al*. (2016) and Reddy *et al*. (2010) demonstrated an absence of any connection between this variable and the intention to use medical tourism services [44,45].

To sum up, as demonstrated by other researchers, attitude proved to be the most important explanatory variable in the TPB model, while subjective norms and perceived behavioral control were less important. Seow *et al*. (2016), in whose study young respondents also dominated, explained the dominant role played by attitude as younger respondents being more eager to experience new things, while the lesser importance of subjective norms was explained by the greater inclination of respondents toward individualism and lower susceptibility to external influences [45]. The smaller importance of perceived behavioral control may result from the relative ease of travel as perceived by young people and a possible lack of understanding of the scale of problems related to the organization of medical travel.

When analyzing the role of the additional factors taken into account by the authors, it can be observed that relations between the variables in the basic TPB model vary significantly depending on the respondents’ country of origin. The strongest impact of COO on the relationship between attitude and behavioral intention was observed in the case of the respondents from Poland, while the weakest impact was observed in the case of the respondents from Turkey; the strongest impact of COO on the relationship between subjective norms and behavioral intention was observed among the respondents from Turkey, while a weaker impact (at a comparable level) was revealed in relation to the respondents from Jordan and Poland. These differences may be caused by social, cultural, legal or economic factors and would require further analysis. One potential explanation is the difference in the level of individualism—the cultural dimension that determines the degree of interdependence a society maintains among its members [82]. Previous studies showed a greater relationship between subjective norms and behavioral intentions in collectivistic rather than individualistic countries [48]. Poland, with an index of 60 points, is an individualistic, and Turkey (with 37 points) is a collectivistic society. Belonging to a group is very important in Turkish society, so group norms are also more important [120]. COO factor also proved to be important in the study by Lubowiecki-Vikuk and Dryglas (2019), where the analysis concerned the differences among respondents examined from various countries in the context of preferred medical tourism services provided in Poland [116].

Similarly to the study by Guy *et al*. (2015) [50], the results of this study did not show any relationship between willingness to consider medical tourism services and gender (different results were obtained by Holliday *et al*. (2015) and Lubowiecki-Vikuk and Dryglas (2019) [116,121]), nor the frequency of international travel declared by the respondents.

This study revealed that declared health condition moderates the manner in which attitude and perceived behavioral control influence behavioral intention in the context of using medical tourism services. The higher the level of self-assessment of the respondents’ health conditions, the stronger the effect of attitude on behavioral intention, and conversely, the weaker the effect of perceived behavioral control on behavioral intention. In the study by Guy *et al*. (2015), no similar results were obtained [50]. However, in both studies, there was a fundamental difference in the formulation of the question pertaining to health condition; namely, in the present study, it concerned the declared health condition of the respondent, while in Guy’s case, the respondents filling in the questionnaire were supposed to imagine that they were in one of three medical conditions: a life-threatening condition, a serious but not life-threatening condition, or a condition considered to be medically optional.

The study furthermore examined the relationship between perceived risk and attitude and the variables that potentially moderate that compound: risk awareness, risk aversion, satisfaction with the domestic service, country of origin, gender, health condition and frequency of foreign travel.

The results of the current research have revealed the strong negative impact of *perceived risk* on *attitude*, which means that the greater the perceived risk, the less favorable the attitude to medical tourism on the part of the respondents. This result is consistent with the results of studies in the MT area conducted by Seow et al. (2017), Liang et al. (2019) and Dash (2021). Quintal *et al*. (2010) obtained similar results using the TPB model; however, they studied tourism services in general, rather than medical tourism in particular, showing a negative effect of varying degrees of *perceived risk* on *attitude* in an analysis of the intention to visit Australia of residents of South Korea and Japan (a significant effect) and China (a directional effect only) [62]. Seow *et al*. (2017) conclude that the negative impact of *perceived risk* on *attitude* “enhances the ability of the attitude in influencing tourists’ behavioral intention” demonstrated in their study [29], p. 389). This conclusion, however, is intuitive in its nature, as the authors have not examined it. It can also be concluded that the obtained result is, in a sense, also consistent with the results of the study by Chelliah et al. (2021) [33].

The results of the present survey indicate that there are no statistically significant differences in the impact of *perceived risk* on *attitude* among respondents from different countries.

In terms of the moderation strength for the relationship between *perceived risk* and *attitude*, the sequence of the factors introduced into the model was as follows (starting with the strongest): *risk awareness*, *risk aversion*, *gender*, and *satisfaction with the domestic service*. *Foreign travel frequency* affects *attitude*, yet without a moderating effect, while *country of origin* and *health condition* as declared by respondents were not significant.

*Risk awareness* was the strongest moderator, and although the direction of its impact was contrary to intuition, it does not change the negative direction of the relationship between *perceived risk* and *attitude*. *Risk aversion*, to a smaller yet still significant extent, formed the relationship between *perceived risk* and *attitude*. Here, the relationship was in line with intuition: Respondents with higher levels of *risk aversion* demonstrated a less favorable attitude to medical tourism when perceiving it as risky than did less risk-averse respondents. This is in line with the results of the study by Kemp *et al*. (2015), which demonstrated differences in risk perception between respondents declaring higher and lower levels of risk aversion in the context of MT respectively [122]. The study conducted by Guy *et al*. (2015) found no relationship between un/willingness (understood as intention) to consider travelling abroad for medical treatment and risk aversion [50].

*Gender* also proved to be a moderating variable, because the relationship between *risk* and *attitude* is more negative for men than for women, which means that, in the case of young men participating in the study, a negative attitude to medical tourism increased as the perceived risk increased more than it did for women. According to the authors’ knowledge, there are no thematically convergent studies in the current scientific discourse whose results could be referred to in this particular area.

It needs to be emphasized that the impact of *risk awareness*, *risk aversion* and *gender* only weakens or strengthens the relationship between *perceived risk* and *attitude*, rather than changing the direction of the relationship, which is always strongly negative.

The research has also demonstrated the significant impact of the *satisfaction with the domestic service* variable on *attitude*. People who assessed medical services in their home country as being of higher quality exhibited a less favorable attitude towards medical tourism. This seems natural given the fact that travelling abroad adds to and reinforces the risks ascribed to the use of medical services in one’s country of residence.

Moreover, the results of the study demonstrated that, similarly to Yuzhanin and Fisher (2016, p. 141)[54], attitude, subjective norms and perceived behavioral control do not exhaust the set of those variables that influence the behavioral intentions of potential medical tourists. The same applies to the model for attitude with perceived risk as one of the predictors.

## Conclusions

The objectives pursued by the authors of the study include an investigation of the role and importance of perceived risk in medical tourism using the extended TPB model. The applicability of TPB for testing the intention to use medical tourism services was verified. The TPB model was extended to include the risks associated with the use of medical tourism services as perceived by potential consumers. Comparative analyses of European and Western Asian countries on the basis of these studies fill the existing research gap in this area.

The analysis of the survey data permitted the verification of the hypotheses put forward, and it demonstrated that the assumptions of the TPB model can be applied in medical tourism. In the segment of young consumers surveyed, all the three explanatory variables in the basic TPB model, i.e. attitude, subjective norms and perceived behavioral control, had a significant impact on the respondents’ intentions to use medical tourism services. The results furthermore demonstrated that the country of origin and the assessment of the respondents’ own health condition significantly moderate the influence of their attitude towards medical tourism on their behavioral intention to use these services.

Furthermore, the epistemic value of the present research may be revealed in the conclusions from the analysis of the extension of the TPB model, which serve to confirm a strong relationship between the risk perceived by the respondents and their attitude towards medical tourism services. The factors that significantly moderate this relationship included the following: risk awareness, aversion to risk, and the respondents’ gender.

### Managerial and policy implications

The results of the study show, among others, the influence of the country of origin on the intention to use medical tourism services, which should be reflected in the geographic profiling of marketing campaigns initiated by medical tourism service providers as well as by those institutions that are responsible for the development of the marketing offer of a given destination (e.g. national and local tourism organizations, national health organizations, ministries of tourism, and individual tourism providers, such as travel agencies or tour operators).

This research has shown that attitude is the most important factor in the TPB model that shapes consumer intentions in medical tourism. Therefore, in the context of creating future demand for the services in question, these entities should undertake activities aimed at building a positive assessment of the expected effects of using MT. This can be communication indicating, for example, the advantages of using MT in comparison with the domestic offer, such as shorter waiting period, more modern services or those that are not available on the domestic market [123]. This communication can also indicate the tourist aspect of such a trip.

This study has also shown that the greater the perceived risk, the less favorable the respondents’ attitude towards using medical tourism. Through an appropriate communication policy regarding, for example, the quality of services, the security of procedures (which could be confirmed by appropriate certificates and accreditations), support in the organization of travel and stay or the possibility of communication in the native language, the service providers may eliminate the sense of risk. However, the necessity of forming the message in a reliable manner needs to be stressed, as the lack of awareness of real risks (which, in light of this research, may significantly affect young people) may give rise to excessive expectations and, as a result, disappointment and dissatisfaction with the service actually received or problems of a medical or legal nature arising as a result. Kemp *et al*. (2015) presented differences in the impact of emotional and rational advertising on perceived risk in MT by people with high and low levels of risk aversion respectively [122]. This may give rise to the temptation to manipulate a potential customer; consequently, one needs to agree with the authors of these studies as to the particular importance of taking ethical standards on the part of those responsible for advertising messages, as well of as the control of state regulation agencies over them, into account.

This research has also shown the important role of social norms in shaping purchasing intentions. This factor reflects the importance of reference groups in the decision-making process of medical tourists [124]. Therefore, it is important to shape a positive image of medical tourism not only in the eyes of the tourists themselves, but also in the society in general, assuming that it will favor the positive opinions of the immediate environment of potential clients regarding the treatment abroad. An important role in supervising the quality of the offer and in PR and publicity activities addressed to the general public should be attributed to the active role of government agencies [125] and industry associations. Furthermore, MT providers could distribute the information materials that potential patients could share in their environment. They could also include the possibility of accompanying patients during their trips and offer the support in the trip organization. Such activities would also increase the behavioral control perceived by medical tourism services users.

In the authors’ opinion, the decision to carry out the research on educated young consumers (future potentially aware medical tourists) creates an additional cognitive value of the research in the context of its usefulness in the process of creating long-term strategies for the functioning and development of entities on the medical tourism services market.

The basic image building element, also in the context of MT, is user experience. If this experience is satisfactory for patients using foreign medical services, they will promote it through positive word-of-mouth communication. Therefore, it is in the best interest of each service provider, as well as the entire industry, to care for the highest standards of services provided and the quality of experience of foreign patients. It should also be noted that the above-mentioned experience includes not only medical services, but also hotel, catering and other services that can be used by tourist patients during their stay.

### Limitations and future studies

Despite the fact that an extension of the TPB model to include perceived risk allows one to explain the variability of behavioral intentions to a greater extent, this study demonstrates that it still does not cover all the relevant variables that have an impact on the behavioral intentions of the potential consumers of medical tourism services. An attempt to identify further model variables could be of cognitive value in future research.

As this research was conducted just before the COVID-19 pandemic outbreak, the research assumptions did not take into account the perceived risk of COVID-19 contagion. As a potentially important variable in the multidimensional risk construct, the perceived risk of COVID-19 contagion by the respondents is undoubtedly a very important direction for further research on this issue. In the context of limitations directly related to the survey process, it should be noted that the sample selection in the survey in question was non-random as it focused on a segment of young consumers (students). Therefore, one needs to be cautious as regards both generalizing the results in relation to the young consumer population examined and to remember not to generalize them in the context of general population as well.

In light of the above, an interesting direction for further research into this aspect of medical tourism could be to extend the area of interest to cover other age groups of potential medical tourists, including those from Jordan, Poland and Turkey, as well as from other countries which have not yet been examined.

In addition to this, carrying out similar research on actual medical tourists from these three countries would also have potentially high cognitive value. This would make it possible to examine the potential intention to continue to use the services of medical tourism once one has gained experience of this type of offer.
